# Diagnostic appraisal of water quality and pollution control realities in Yasu River using GIS-aided epsilon robust optimization model

**Published:** 2004-08-01

**Authors:** Toshihiko Kawachi, Shigeya Maeda

**Affiliations:** Graduate School of Agricultural Science, Kyoto University, Kitashirakawa Oiwake-cho, Sakyo-ku, Kyoto 606-8502

**Keywords:** Diagnostic appraisal, optimization, Geographic Information System, wasteload allocation, river water quality management, uncertainty

## Abstract

Realities of in-stream water quality and its control in Yasu River, Japan are diagnosed by use of an epsilon robust optimization (*ε*-RO) model with the aid of a Geographic Information System (GIS). The GIS helps delineate catchment boundaries associated with a diagnosed section of the river, find outfalls of wastewater, and estimate both discharge and uncontrollable COD (Chemical Oxygen Demand) load from nonpoint sources. Twelve hydro-environmental scenarios assumed to stochastically occur in a year are generated based on historical data. Calibration of a finite element model for COD and dissolved oxygen transport is conducted to identify some scenario components. The *ε*-RO model determines the optimal wasteload quotas at point sources (PSs) through allocation of the maximized total COD load, which lead to ameliorating water quality in the river. The diagnostic results show that there is every possibility that the in-stream water quality is upgraded even if total COD loading is increased so long as it is appropriately allocated to PSs.

## Introduction

An optimization model for COD (Chemical Oxygen Demand) load allocation in rivers, *ε*-RO (epsilon robust optimization) model, was described in the companion paper.1) In this paper, a modified *ε*-RO model, where COD and DO (Dissolved Oxygen) transport equations are slightly changed and strict constraints on river water quality at the downstream end of a river are added, is applied to a part of Yasu River in Shiga prefecture, Japan, in order to diagnose the current COD loading by comparing it with the noninferior solutions obtained. A Geographic Information System (GIS), ArcView GIS with Spatial Analyst module, is employed to delineate catchment boundaries in the river basin, and to identify the locations of outfalls along the river, where wastewaters from point sources (PSs) and nonpoint sources (NPSs) are virtually injected. Amounts of COD load from NPSs are evaluated on the GIS by using land use map and unit loading factor (ULF). Optimization practices show that there is every possibility that river water quality is ameliorated even with the increase in total COD loading.

## Study area and diagnosed river section

**Fig. 1:**
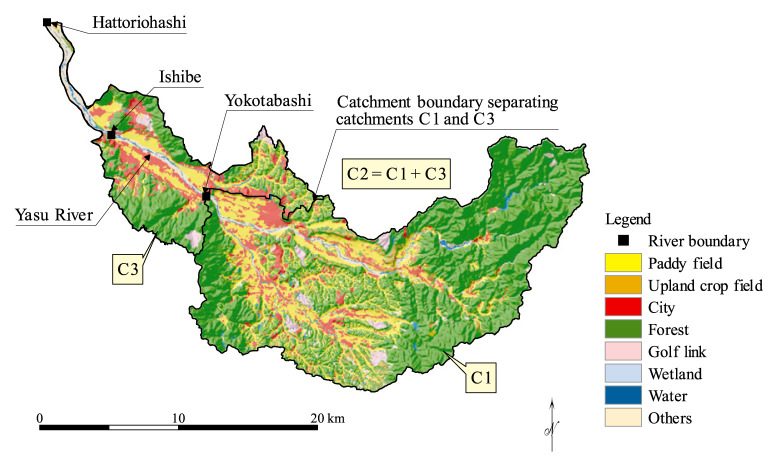
Yasu River basin whose outlet exists at Hattoriohashi.

**Fig. 2:**
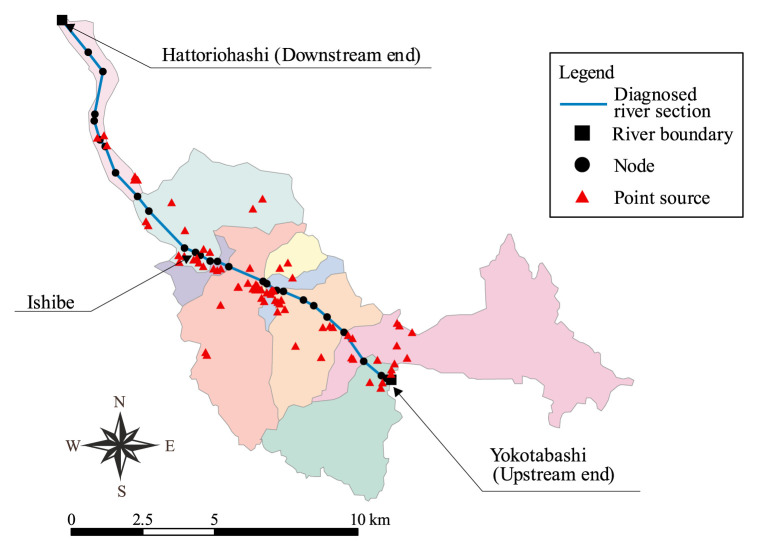
Diagnosed river section, point sources and subcatchments.

Yasu River is one of the major rivers feeding into Lake Biwa, Shiga Prefecture, Japan. The overall drainage of the river is 61km long, and its entire catchment is 387km^2^ wide. In this study, a downstream part of the river, 18.6 km long from Yokotabashi to Hattoriohashi, is considered as a “diagnosed river section” where water quality and its control realities are diagnostically appraised in contrast with optimal realizations. The subcatchments of interest are those having the upstream and downstream ends (Yokotabashi and Hattoriohashi, respectively) of the diagnosed section as their outlets. The respective subcatchments, C1 and C2, and their drainage divides detected by use of GIS are shown in Fig. 1. The subcatchment from that surface runoff and/or drainage water flow laterally into the diagnosed section, C3, can thus be obtained by removing C1 from C2. This extracted subcatchment C3 of 89.6 km^2^ wide, hereinafter called catchment system, is redrawn in Fig. 2 that also marks the locations of PSs interspersed over this system.

The catchment system can further be fragmented into nine subcatchments (Fig. 2). These are delineated using a digital elevation model (DEM) with a grid resolution of 50m × 50 m, provided by the Japan Geological Survey Institute and a traditional topographic map. Drainage networks, which are permanent or temporal stream channels, in the catchment system are also identified using the GIS based on the DEM. As a result of such on-map drainage-routing identification, the mouths of tributaries feeding the diagnosed section can also be identified. Nodal points are individually assigned to the outlets of subcatchments and to the mouths of tributaries.

Wastewaters discharged from both PSs and NPSs are considered. Within the catchment system, there are 74 industrial plants to be considered as major PSs. Their effluent discharges and COD concentrations are known. Wastes from 74 original sources are virtually loaded at 14 loading points (nodes) along the section. Lateral discharges from NPSs, which are *a priori* unknown, can be deduced from gross water balance in the subcatchment C3. NPS-born wastewaters diffused from a subcatchment can be assumed to collect at its outlet, and therefore all of 9 subcatchment outlets are taken as loading points of NPS-born wastewaters.

**Fig. 3:**
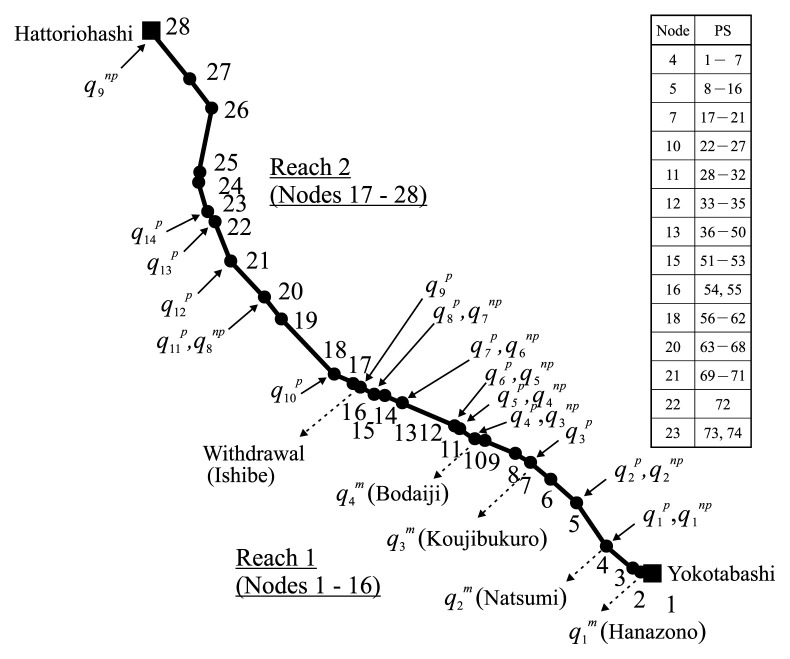
Inflow/outflow locations, and virtual loading points (nodes) of PS-born wastewaters.

For numerical representations of stream flow and substance (COD and DO) transport in the river, the diagnosed section is piecewise discretized into 27 line elements with 28 nodes, as depicted in Fig. 3. The diversion weir located at Ishibe, from which river water is withdrawn for irrigation use, rapidly and significantly changes flow and transport in the river. The section is, therefore, divided into two reaches: Reach 1 of 7.8 km long (Nodes 1 to 16), and Reach 2 of 10.6 km long (Nodes 17 to 28). Reach 2 is separated from Reach 1 with distance of 0.2 km. These two reaches are, however, virtually linked through appropriate functional relations on flow and transport between different two Nodes 16 and 17 that are treated as internal boundaries in numerical representation.

Paddy field irrigation for rice cropping is practiced through fetching water from the river. There are 5 of irrigation water withdrawals altogether that significantly affect flow and water quality realizations in the river, including the one at Ishibe diversion weir. Withdrawals at the locations indicated as Hanazono, Natsumi, Koujibukuro and Bodaiji in Fig. 3 are thus treated as lateral outflows from their respective nodal points, whereas withdrawal at Ishibe (Node 16) is considered as a discharge reduction at Node 17.

## Scenarios

The *ε*-RO model requires scenario-based description of hydro-environmental input data or parameters to reflect their uncertainties to noninferior solutions. The authors1) addressed that, for the problem envisaged, considering over-the-year hydroenvironmental uncertainties on a monthly basis could provide plausible scenarios. According to this, a set of twelve scenarios is generated in that the respective scenarios have probabilities of occurrence in proportion to the throughout-the-month number of days. Scenario components encapsulated in a scenario, i.e., hydro-environmental input data or parameters, are evaluated based on the recorded or observed historical data available.

## COD and DO transport equations

The equations describing COD and DO transport in rivers belong to the central core of the constraints in our *ε*-RO model. In the present case that is rather general, their original mathematical expressions in an ordinal differential form must be modified to include additional non-conservative terms that express substance losses resulting from irrigation water withdrawals. Thus the associated constraints must be provided based on the following form of the equations.

[1] $$Q\frac{dL}{dx}-\frac{d}{dx}\;\left(AD_{x}\frac{dL}{dx}\right)+AK_{1}L+\left(\sum_{k}{\bar{q_{k}}}^{p}+{\bar{q}}^{np}-{\bar{q}}^{m}\right)\;L-\sum_{k}{\bar{q_{k}}}^{p}L_{k}^{p}={\bar{q}}^{np}L^{np}$$

[2] $$Q\frac{dC}{dx}-\frac{d}{dx}\;\left(AD_{x}\frac{dL}{dx}\right)+AK_{1}L-AK_{2}(C_{S}-C)+\left(\sum_{k}{\bar{q_{k}}}^{p}+{\bar{q}}^{np}-{\bar{q}}^{m}\right)\;C=0$$

where the superscripts *p* and *np* denote PS and NPS, respectively, the subscript *k* denotes identification number of PS, *x* = horizontal distance along the river (m), *Q* = cross-sectional discharge (m^3^·s^−1^), *A* = cross-sectional area (m^2^), *L* and *C* = concentrations of COD and DO in main stream water, respectively (mg·L^−1^), $L_{k}^{p}$ and *L^np^* = COD concentrations in laterally injected PS- and NPS-born wastewaters, respectively (mg·L^−1^), ${\bar{q_{k}}}^{p}$ and *q̄^np^* = lateral discharges per unit width issuing from PS and NPS, respectively (m^2^·s^−1^), *q̄^m^* = discharge withdrawn from the river per unit width (m^2^·s^−1^), *D_x_* = longitudinal dispersion coefficient (m^2^·s^−1^), *K*_1_ = deoxygenation coefficient (s^−1^), *K*_2_ = reaeration coefficient (s^−1^), and *C_S_* = saturation value of DO (mg·L^−1^). Following our precedent, eqs.[1] and [2] are transformed by the finite element method into numerical versions that can be embedded in the *ε*-RO as equity constraints. Note that $L_{k}^{p}$ is a controllable decision variable in a sense of optimization, while *L^np^* is a given variable considered as uncontrollable.

## Outflows

Water withdrawal at Ishibe diversion weir varies in time within the range of 0 to 7.32m^3^·s^−1^ (recorded during 1990 to 1998). The remaining four withdrawals at Hanazono, Natsumi, Koujibukuro and Bodaiji are made by pumping during the irrigation period of April to August or September, with mostly invariable discharges of $q_{1}^{m}=0.276\;({\text{m}}^{3}\cdot {\text{s}}^{-1}),q_{2}^{m}=0.598\;({\text{m}}^{3}\cdot {\text{s}}^{-1}),q_{3}^{m}=0.650\;({\text{m}}^{3}\cdot {\text{s}}^{-1}),q_{4}^{m}=0.440\;({\text{m}}^{3}\cdot {\text{s}}^{-1})$, respectively (Fig. 3).

## Wastewater from point sources

PS-born wastewater discharges $q_{k}^{p}$ and their COD concentrations $L_{k}^{p}\;(k=1,\ldots ,74)$ are needed for identifying *K*_1_ and *K*_2_ in eqs.[1] and [2] as well as for knowing COD loading realities in the section. These can easily be computed on a monthly average from the daily-observed data during 1993 to 1995 of fiscal year, which are available at 74 industrial plants.

## Wastewater from nonpoint sources

NPS-born COD loading from every particular land is assumed uncontrollable and fixed, its amount being a product of the corresponding ULF and the area. Different ULFs2) that Shiga Prefectural Government authorizes are used for paddy field, upland crop field, city, forest and golf link. The result of GIS-aided estimate of COD loadings from the respective subcatchments is summarized in Table I.

On the other hand, NPS-born wastewater discharges $q_{j}^{np}\;(j=1,\ldots ,9)$ (Fig. 3) are considered uncontrollable but uncertain. This necessarily indicates that their COD concentrations are uncertain as well. The discharges $q_{j}^{np}$ can be estimated through allotting the gross monthly effluent discharge $Q_{C3}^{np}$ from NPSs in the catchment system C3 among nine subcatchments in proportion to their areas. $Q_{C3}^{np}$ can be computed from the monthly-based gross water balance analysis for C3 (using inflows and outflows shown in Fig. 3, and measured discharges at Yokotabashi and Hattoriohashi).

## Flow analysis

Flow analysis to obtain discharge and water depth at every particular node is a need prior to operation of the optimization model. First, nodal discharges are calculated downstream from Yokotabashi considering in- and outflows along the river course. Flow profile is then step-by-step computed upstream by the steps from node to node to obtain water depths along the course in each reach, using the standard trial-and-error step method for gradually varied flow analysis. The initial water depth from which the computation is started is taken as the depth resulting from uniform flow approximation at the downstream end of the reach.

## Calibration of COD and DO transport model

Determination of physical constraints of COD and DO transport further requires estimating values of *D_x_*, *K*_1_, *K*_2_ and *C_S_* in eqs.[1] and [2]. *D_x_* and *C_S_* are estimated by use of the well-suited empirical equations.1) *K*_1_ and *K*_2_ are identified for each of the two reaches (Reaches 1 and 2) through calibrating the finite element model for COD and DO transport equations. The model is run under the boundary conditions; Dirichlet condition at the upstream end and Neumann condition (null flux) at the downstream end for both COD and DO transports. Thus their values are tuned in a trial-and-error fashion so that the computed COD and DO values at the downstream end of the reach may coincide with the observed ones. However, since the observed data of COD and DO at the downstream end (Node 16) of Reach 1 are not available, they are deduced from the data available at the upstream end (Node 17) of Reach 2. COD at Node 16 is presumed to remain unchanged while going downstream through the weir, and therefore to be identical with that at Node 17. Meanwhile, DO at Node 16 is, due to oxygen supply by aerated nappe overflowing the weir, increased up to that at Node 17. To take this effect into account, DO value at Node 16 is, based on the following well-established formula,3) deduced from known DO value at Node 17.

[3] r=1+0.38abH(1-0.11H)(1+0.046T)

where *r* = ratio of DO deficit just before and after the weir, *H* = difference in water elevation (m), *T* = water temperature (°C) and *a* and *b* = 1.0 and 0.80, respectively.

## Optimization

As described above, a set of twelve scenarios is provided to derive optimal water pollution control strategies under hydroenvironmental uncertainties. Here, since *Q*, *L* and *C* at the upstream end of reach, *h* at the downstream end of reach, $q_{j}^{np}\;(j=1,\ldots ,9)$, *T*, *K*_1_ and *K*_2_ are uncertainly variable, these are considered as the scenario components and quantified for each scenario through statistically manipulating their observed historical data (Table II). The lower limit of effluent COD concentration is taken as $L_{js}^{pl}\;=0.0\;(\text{mg}\cdot {\text{L}}^{-1})$. The upper limit of the concentration, $L_{js}^{pu}$, is determined for each PS, considering the nationwide applied effluent standard (ES) and the local stringent add-on effluent standard (AES) (only for wastewater whose discharge $q_{k}^{p}\geq 10\;{\text{m}}^{3}\cdot {\text{day}}^{-1}$) which are provided by the Water Pollution Control Law and the prefectural bylaw, respectively. Note that different AESs are provided for different industries and different effluent discharges which are properly classified and ranked, respectively. As by the Basic Environment Law enacted, $C_{is}^{ol}=7.5\;\text{mg}\cdot {\text{L}}^{-1}$ is employed as the lower limit of in-stream DO concentration. The law provides the COD- and BOD-based organic pollutant limits for lakes and rivers, respectively. The COD-based limit for Yasu River is therefore induced from that for Lake Biwa, through apportioning the pollutants, allowed to load during the gross water residence time in the lake, among the individual river basins. The COD concentration apportioned to Yasu River, $L_{Y}^{u}$, is thus estimated by eq.[4] to have the upper limit of in-stream COD concentration, $L_{is}^{ou}\;(=L_{Y}^{u})=2.3\;(\text{mg}\cdot {\text{L}}^{-1})$.

[4] $$L_{Y}^{u}=\left(\frac{V_{B}}{Q_{Y}T_{B}}\right)\;\left(\frac{A_{Y}}{A_{B}}\right)L_{B}^{u}$$

where $L_{B}^{u}$ = allowable limit of COD concentration in Lake Biwa (= 1 mg·L^−1^), *V_B_* = storage volume of Lake Biwa (= 2.75 × 10^10^ m^3^), *A_Y_* = area of Yasu River basin (= 387 km^2^), *Q_Y_* = inflow from Yasu River (= 2.44 m^3^·s^−1^ calculated from monthly observed data at Hattoriohashi in 1983–1999), *T_B_* = water residence time in Lake Biwa (18.9 years4) estimated by considering mixing of inflowing river water with the pre-existing lake water), and *A_B_* = catchment area of Lake Biwa (= 3,174 km^2^).

Two new constraints, expressed as eq.[5], are added to explicitly control water quality at the downstream end of the section with the intention of mitigating water quality deterioration in Lake Biwa.

[5] $$L_{s}^{d}\leq L_{s}^{du},\;\;\;C_{s}^{d}\geq C_{s}^{dl},\;\;\;\forall s$$

where $L_{s}^{d}$ and $C_{s}^{d}=\text{COD}$ and DO concentrations at the downstream end of the section, respectively, $L_{s}^{du}=\text{upper limit for }L_{s}^{d}$, and $C_{s}^{dl}=\text{lower limit for }C_{s}^{d}$. The limits, $L_{s}^{du}$ and $C_{s}^{dl}$, are now identified with the averages of monthly observed COD and DO concentrations at Node 28, respectively, with a view to keeping at the very least the current level of water quality at the downstream end of the section.

The *ε*-RO model leaves the prime objective *f*_1_ in the objective function as it is, and adapts the remaining objectives *f*_2_, *f*_3_ and *f*_4_ to the constraints. All those component objectives, defined in our last paper,1) are the criteria to be minimized. The *ε*-RO model presently considered thus requires

[6] $$\text{Minimize }\;\;f_{1}(\cdot )$$

subject to the *ε*-constraints

[7] $$f_{j}(\cdot )\leq {ɛ}_{j},\;\;\;j=2,\;3,\;4,$$

eq.[5] and other essential constraints.1)

Use of the *ε*-RO model must be preceded by determination of *ε_j_* that binds *f_j_*(·). Here, minimums of *f_j_*(·) (*j* = 2, 3, 4) are found to be 0.000 g·s^−1^, 2.571 mg·L^−1^ and 33.857 mg·L^−1^, respectively. In order to produce feasible noninferior solutions, *f_j_*(·) are actually bound by *ε_j_* larger than their minimums obtained.

## Results and discussion

The *ε*-RO model presented here is solved by the simplex method to obtain the noninferior solutions *<*Solution A*>* and *<*Solution B*>*. *ε*_2_ = 0.11 is selected in common to both solutions. Difference is made for other *ε*-constraints, taking (*ε*_3_, *ε*_4_) = (5.57, 34.0) for *<*Solution A*>* and (*ε*_3_, *ε*_4_) = (3.60, 34.4) for *<*Solution B*>*. Since *f*_2_(·) is bound by a relatively small *ε*_2_ (= 0.11), both solutions are those which are so robust or insensitive to hydro-environmental uncertainties as to be normative.

**Fig. 4:**
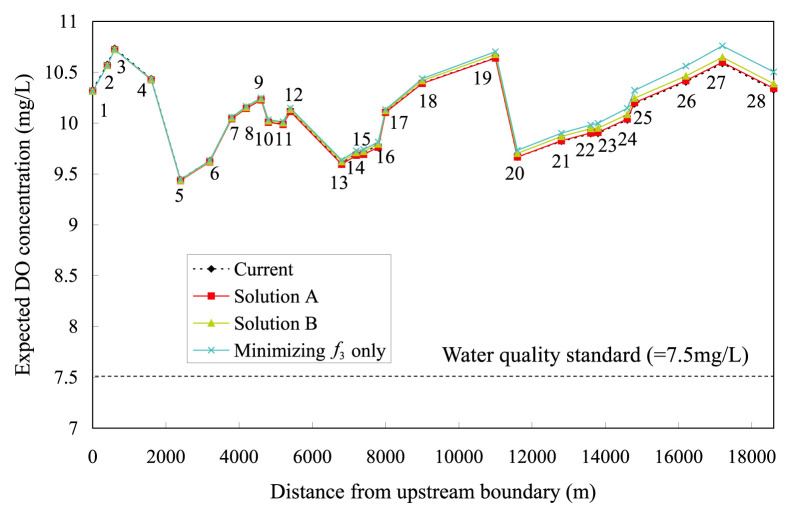
Expected DO concentration profiles (1–28 along line are node numbers).

**Fig. 5:**
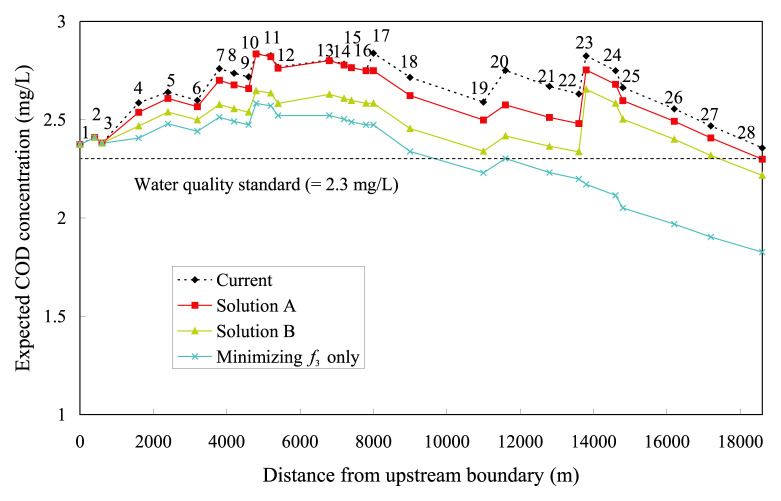
Expected COD concentration profiles (1–28 along line are node numbers).

Profiles of the expected in-stream DO and COD concentrations, for both *<*Solution A*>* and *<*Solution B*>*, are illustrated in Fig. 4 and Fig. 5, respectively. The figures also include other two profiles. One is the current DO or COD profiles to be diagnosed, deduced by simple transport simulations. The other is the expected DO or COD profiles derived by solving a single-objective problem that only takes *f*_3_ as the objective function, which no longer intends maximizing or increasing the total COD load. This COD profile is that for a limit to controlling in-stream COD concentrations, entailing an extreme reduction of the total COD load from the current 4.694 g·s^−1^ to the expected 1.265 g·s^−1^. Fig. 4 indicates that the reallocations of COD load based on *<*Solution A*>* and *<*Solution B*>* will bring no drastic change to the current in-stream DO concentration.

In *<*Solution A*>*, water pollution could be alleviated with perceptible COD reduction (Fig. 5). The COD concentration then averages 2.611 mg·L^−1^ per one node, which is slightly smaller than the current value of 2.662 mg·L^−1^. In *<*Solution B*>*, due to the smaller value of *ε*_3_, the COD concentration is effectively lowered with less violated deviations from its standard, averaging 2.493 mg·L^−1^ per one node. *<*Solution B*>* is therefore preferable to *<*Solution A*>* in terms of in-stream water quality amelioration. Fig. 5 also says that the current COD concentration at the downstream end of the diagnosed river section violates the desirable condition at the mouth of Yasu River (2.3mg·L^−1^ estimated by eq.[4]), while both *<*Solution A*>* and *<*Solution B*>* satisfy it.

In terms of gross allowable PS-born COD load, *<*Solution A*>* could provide a better alternative loading strategy, permitting a total load of 4.718 g·s^−1^ more than 4.694 g·s^−1^ currently loaded. On the other hand, *<*Solution B*>* provides an inferior alternative, permitting the load of 3.621 g·s^−1^ in total.

**Fig. 6:**
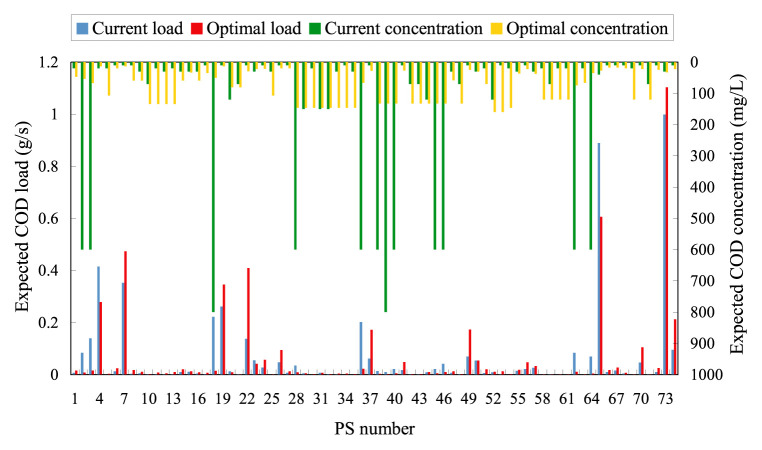
Optimal PS-born COD loads and wastewater qualities in COD (*<*Solution A*>*).

Whichever solution is preferred, it is needed to strategically allocate the expected total COD load among PSs according to the COD limits allowed for individual wastewaters. Such COD limits are those to be considered as the alternatives to the currently applied ES or AES. Clearly, interdependencies among PSs as well as individual industry- and discharge-dependencies are reflected in these limits. Fig. 6 illustrates the optimal quotas of wasteload at PSs and the resulting allowable limits of COD concentration in individual wastewaters for *<*Solution A*>*. In this case, 19 PSs out of 74 are required to upgrade their wastewater qualities to the respective allowable COD levels. Meanwhile, in *<*Solution B*>*, such quality upgrading is required at 33 PSs more than in *<*Solution A*>* because of the decreased total COD load.

## Conclusions

Using the *ε*-RO model with the aid of ArcView GIS, the water quality and pollution control realities in Yasu River have been diagnosed. As the diagnostic results show, there are some management alternatives for upgrading the water quality of Yasu River, which could lead to the water quality restoration of Lake Biwa, including at least one that allows to increase the PS-born wasteload in total. Of further interest is the relatively high COD levels in the upstream half of the river section, which are found even in controlled COD profiles. These are undoubtedly due to NPS-born wasteloads that were assumed uncontrollable. It is thus considered that, for more effective water pollution control, effluent controls must range over the agricultural and/or urban sectors. Development of an advanced model that treats such effluents as controllable is straightforward.

Any recommended change for the better entails reallocating the wasteload, subsequently forcing some of the local dischargers to decrease COD loading. Since, as stated in our last paper,1) such any alternative is that from supervisory optimization with no consideration of cost-effectiveness, local optimizations with economic and social facets may further be needed for dischargers to practically take necessary actions.

## Figures and Tables

**Table I:** NPS-born COD loadings

Subcatchment *j*	Node	COD loading$q_{j}^{np}L_{j}^{np}\;(\text{g}\cdot {\text{s}}^{-1})$

1	4	0.9556
2	5	2.5740
3	10	0.9771
4	11	0.1978
5	12	0.1944
6	13	1.6259
7	15	0.2054
8	20	1.1887
9	28	0.0910

**Table II:** Key parameters of scenarios (Min.: Minimum; Max.: Maximum; *R*1: Reach 1; *R*2: Reach 2)

Reach 1		Min.	Max.	Mean
Yokotabashi	*Q*_1_ (m^3^·s^−1^)	3.96	11.57	6.80
	*L*_1_ (mg·L^−1^)	1.77	3.14	2.37
	*C*_1_ (mg·L^−1^)	8.54	12.41	10.33

Ishibe	*h*_16_ (m)	0.37	0.61	0.47

Subcatchment outlets	$q_{1}^{np}\;({\text{m}}^{3}\cdot {\text{s}}^{-1})$	0.0394	0.9407	0.4158
	$q_{2}^{np}\;({\text{m}}^{3}\cdot {\text{s}}^{-1})$	0.0756	1.8030	0.7969
	$q_{3}^{np}\;({\text{m}}^{3}\cdot {\text{s}}^{-1})$	0.0328	0.7831	0.3461
	$q_{4}^{np}\;({\text{m}}^{3}\cdot {\text{s}}^{-1})$	0.0062	0.1481	0.0655
	$q_{5}^{np}\;({\text{m}}^{3}\cdot {\text{s}}^{-1})$	0.0070	0.1679	0.0742
	$q_{6}^{np}\;({\text{m}}^{3}\cdot {\text{s}}^{-1})$	0.0522	1.2457	0.5506
	$q_{7}^{np}\;({\text{m}}^{3}\cdot {\text{s}}^{-1})$	0.0064	0.1532	0.0677

Whole reach	*T^R^*^1^ (°C)	5.2	25.9	15.5
	$K_{1}^{R1}\;({\text{day}}^{-1})$	0.00	11.5	3.00
	$K_{2}^{R1}\;({\text{day}}^{-1})$	0.00	3.41	2.01

Reach 2		Min.	Max.	Mean
Ishibe	*Q*_17_ (m^3^·s^−1^)	1.66	12.42	5.35
	*L*_17_ (mg·L^−1^)	2.24	3.64	2.84
	*C*_17_ (mg·L^−1^)	7.88	12.02	10.11

Hattoriohashi	*h*_28_ (m)	0.26	0.58	0.39

Subcatchment outlets	$q_{8}^{np}\;({\text{m}}^{3}\cdot {\text{s}}^{-1})$	0.0416	0.9920	0.4385
	$q_{9}^{np}\;({\text{m}}^{3}\cdot {\text{s}}^{-1})$	0.0141	0.3358	0.1484

Whole reach	*T^R^*^2^ (°C)	5.4	27.1	16.0
	$K_{1}^{R1}\;({\text{day}}^{-1})$	1.47	9.02	4.57
	$K_{2}^{R1}\;({\text{day}}^{-1})$	0.65	3.20	1.63
